# A satellite-derived dataset on vegetation phenology across Central Asia from 2001 to 2023

**DOI:** 10.1016/j.dib.2024.110297

**Published:** 2024-03-06

**Authors:** Chao Ding

**Affiliations:** a Department of Geographic Science, Faculty of Arts and Sciences, Beijing Normal University, Zhuhai 519087, China; b Center for Territorial Spatial Planning and Real Estate Studies, Beijing Normal University, Zhuhai 519087, China

**Keywords:** Land surface phenology, Ecosystem dynamics, Drylands, Climate change, Remote sensing, MODIS, Near-infrared reflectance of vegetation

## Abstract

Satellite-observed land surface phenology (LSP) data have helped us better understand terrestrial ecosystem dynamics at large scales. However, uncertainties remain in comprehending LSP variations in Central Asian drylands. In this article, an LSP dataset covering Central Asia (45–100°E, 33–57°N) is introduced. This LSP dataset was produced based on Moderate Resolution Imaging Spectroradiometer (MODIS) 0.05-degree daily reflectance and land cover data. The phenological dynamics of drylands were tracked using the seasonal profiles of near-infrared reflectance of vegetation (NIRv). NIRv time series processing involved the following steps: identifying low-quality observations, smoothing the NIRv time series, and retrieving LSP metrics. In the smoothing step, a median filter was first applied to reduce spikes, after which the stationary wavelet transform (SWT) was used to smooth the NIRv time series. The SWT was performed using the Biorthogonal 1.1 wavelet at a decomposition level of 5. Seven LSP metrics were provided in this dataset, and they were categorized into the following three groups: (1) timing of key phenological events, (2) NIRv values essential for the detection of the phenological events throughout the growing season, and (3) NIRv value linked to vegetation growth state during the growing season. This LSP dataset is useful for investigating dryland ecosystem dynamics in response to climate variations and human activities across Central Asia.

Specifications Table

**Table:** 

Subject	Environmental Science
Specific subject area	Global change, terrestrial ecosystem dynamics, remote sensing in ecology
Data format	Analyzed
Type of data	Image
Data collection	This land surface phenology (LSP) dataset was generated using Earth observation satellite data, namely Moderate Resolution Imaging Spectroradiometer (MODIS) surface reflectance and land cover data. The collected time-series satellite images were processed pixel-by-pixel through the following steps: (1) identifying low-quality observations; (2) smoothing the time series; and (3) retrieving LSP metrics. Processing was implemented using Python and MATLAB codes.
Data source location	The spatial coverage of the LSP dataset is 45–100°E, 33–57°N. The MODIS reflectance and land cover data used to generate this LSP dataset can be downloaded from https://search.earthdata.nasa.gov/search
Data accessibility	Repository name: Mendeley data Data identification number: 10.17632/frvnt3jj4w.1 Direct URL to data: https://data.mendeley.com/datasets/frvnt3jj4w/1

## 1 Value of the Data

• The LSP dataset presented herein can be utilized to examine the dynamics of drylands and their responses to climate variation/change and human activities across Central Asia.
• The spatio-temporal dynamics of the LSP metrics are valuable for evaluating ecosystem functions in Central Asia.
• This dataset supports studies concerning the United Nations Sustainable Development Goal (SDG) 15 [1], which is related to ecosystem productivity, biodiversity, and land degradation.

## 2 Background

Information on land surface phenology (LSP) can facilitate our understanding of land surface dynamics [2,3]. Knowledge of LSP has been significantly enhanced by the rapid growth of time-series remote sensing data [3]. Nonetheless, a substantial knowledge gap regarding LSP in Central Asia, a region characterized by extensive drylands, remains [3,4]. The degradation of ecosystem functions and services in drylands is a barrier to sustainable development in Central Asia [5]. Data on LSP are important for comprehending the dynamics of several key ecological indicators related to SDG 15 [6]. Although global LSP products are available, information on LSP in Central Asia remains insufficient, partially due to widespread missing data for sparse vegetation and uncertainties in modeling complex vegetation index (VI) time series [7]. In this context, I developed an LSP dataset specific to the drylands in Central Asia [8] by utilizing satellite-derived images of the near-infrared reflectance of vegetation (NIRv) [9], considering the low vegetation cover and complexity of satellite VI time series in drylands.

## 3 Data Description

The spatio-temporal characteristics of the LSP dataset for Central Asia (LSPCA) are outlined in Table 1. These LSP metrics were categorized into the following three groups: (1) timing of key phenological events, (2) NIRv values essential for detecting the phenological events throughout the growing season, and (3) NIRv value linked to vegetation growth states during the growing season (Table 2). Additionally, other phenological metrics, such as the length of the vegetation growing season (LOS), can be calculated using the provided LSP metrics.

**Table 1:** Spatio-temporal characteristics of the land surface phenology dataset for Central Asia.

Geographic coverage	45–100°E, 33–57°N
Spatial resolution	0.05°
Temporal extent	2001–2023
Temporal resolution	Yearly
Spatial coordinate system	WGS 84 geographic coordinate system

**Table 2:** Characteristics of the land surface phenology metrics.

LSP group	LSP metric	Definition	Value domain
Timing of key phenological events	SOS	Start of the vegetation growing season	-59 to 257
	POS	Maximum peak of the vegetation growing season	32 to 258
	EOS	End of the vegetation growing season	33 to 365
NIRv values essential for detecting the phenological events	NIRv_PEAK	Maximum peak in the NIRv time series	150 to 6000 (actual value: 0.015 to 0.6)
	NIRv_MINL	Minimum of the NIRv time series before the POS	0 to 6000 (0 to 0.6)
	NIRv_MINR	Minimum of the NIRv time series after the POS	0 to 6000 (0 to 0.6)
NIRv value linked to vegetation growth state	NIRv_MEAN	Mean of the NIRv time series from the SOS to the EOS	0 to 6000 (0 to 0.6)

The images of the LSP metrics are included in the compressed file ‘*LSPCA.zip’*. Each LSP metric is stored in an individual subfolder, and the name of the subfolder is the same as that of the LSP metric. Each subfolder contains 23 images. The name of an image denotes the LSP metric and the corresponding year. For example, ‘SOS_2022.tif’ indicates the SOS image for 2022.

**Fig. 1:**
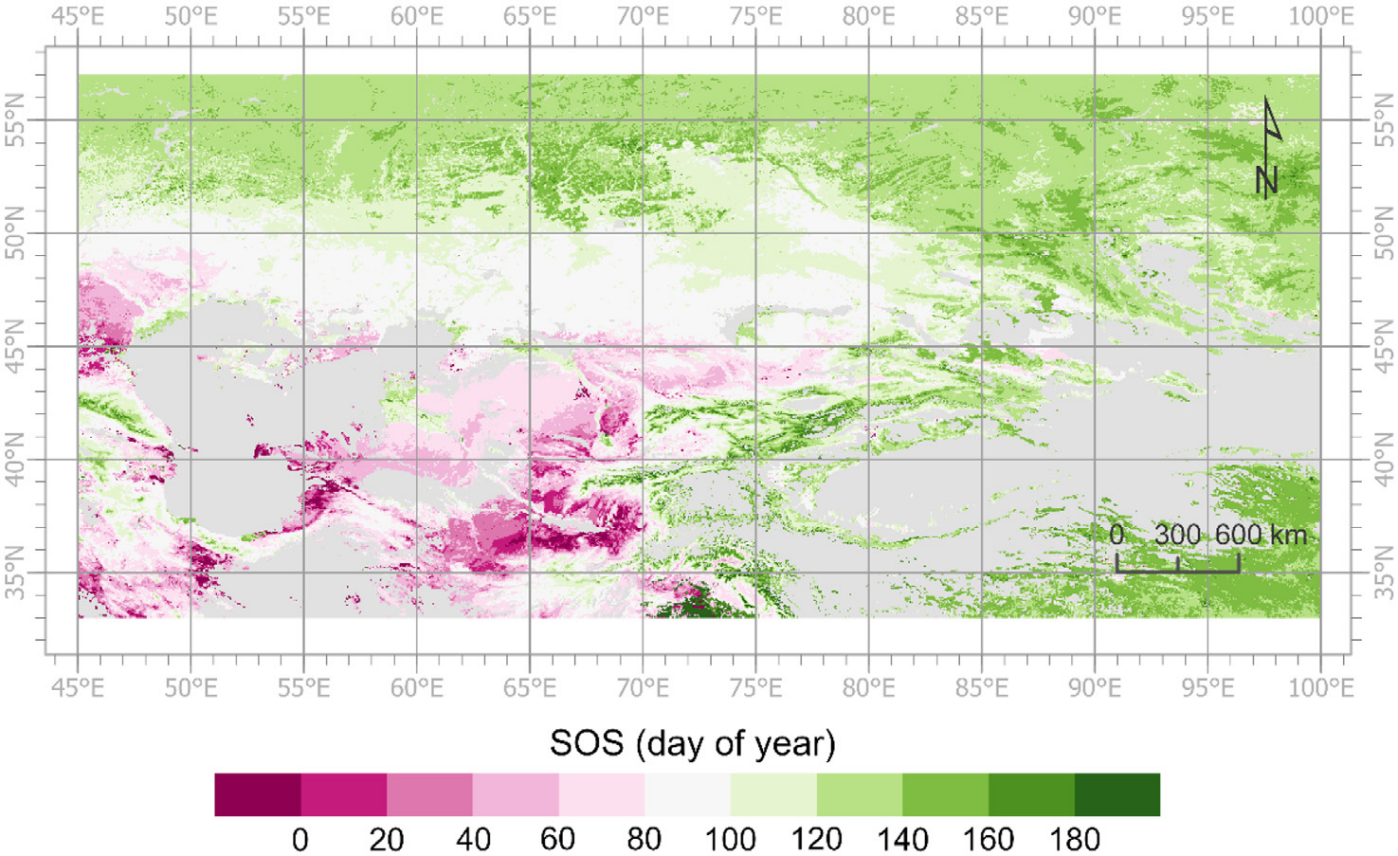
Spatial distribution of the SOS in 2022 derived from the land surface phenology dataset for Central Asia.

**Fig. 2:**
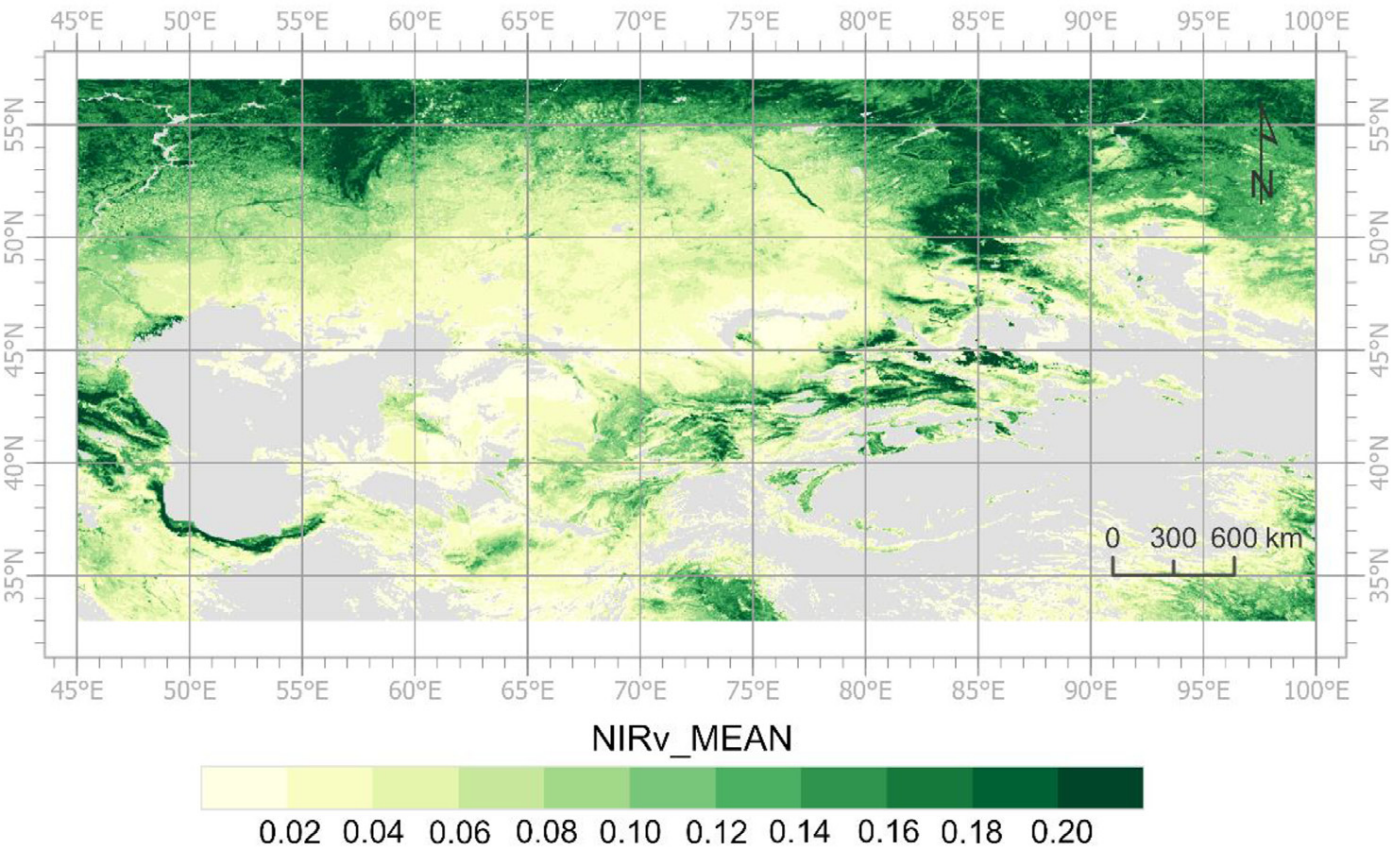
Spatial distribution of the NIRv_MEAN in 2022 derived from the land surface phenology dataset for Central Asia.

The values of POS, SOS, and EOS fall within specific valid ranges, as outlined in Table 2. The POS is within the range of day of year (DOY) 32 to 258. In the southern regions of Central Asia, the SOS may occur during the last two months of the year. For example, the SOS of a pixel has been recorded on 12/9/2021. In this case, the year of the SOS is also identified as 2022, given that the peak NIRv of this growing season occurs in 2022 [10]. Consequently, the SOS value is negative (-22 for SOS on 12/9/2021). The spatial pattern of the SOS in 2022, as depicted in Fig. 1, illustrates numerous instances of negative SOS values. The yearly identifiers for all other LSP metrics are likewise assigned according to the POS [10]. This process allows users to track an entire growing season across two calendar years [10]. Notably, NIRv_MEAN represents the mean NIRv value from the SOS to the EOS, rather than the mean value over a calendar year. Fig. 2 shows the spatial distribution of NIRv_MEAN during the growing season identified as 2022. Additionally, the values of the masked pixels are set at -20,000.

## 4 Experimental Design, Materials and Methods

The MODIS MCD43C4 V061 0.05° daily reflectance data [11] for the period of 2000–2023 and the MCD12C1 V061 land cover data [12] for 2001 were obtained from https://search.earthdata.nasa.gov/search. The NIRv was calculated to track the vegetation seasonality dynamics (Eq. (1)).

(1) $$\text{NIRv}=\left(\frac{\rho_{\text{NIR}}-\rho_{\text{red}}}{\rho_{\text{NIR}}+\rho_{\text{red}}}-0.08\right)*\rho_{\text{NIR}}$$

where $\rho_{\text{NIR}}$ and $\rho_{\text{red}}$ are the surface reflectance values of the near-infrared (Band 2) and red (Band 1) bands of the MODIS sensor, respectively.

NIRv is suitable for observing vegetation dynamics in drylands with a low vegetation cover [9,13]. A previous study reported that NIRv can be used to effectively estimate the gross primary productivity of vegetation in sparse drylands [13].

The following steps were conducted to process the NIRv time series of a land pixel (as identified by the land cover data): identifying low-quality observations, smoothing the NIRv time series, and retrieving the LSP metrics from the smoothed NIRv time series.

### 4.1 Identifying Low-Quality Observations

Observations with a Bidirectional Reflectance Distribution Function (BRDF) quality value greater than 3 were considered to be low quality [14]. Additionally, data with NIRv < 0 and NIRv > 0.6 were identified as low-quality data. The corresponding data points were excluded from the NIRv time series. The normalized difference snow index (NDSI) [15] was employed to detect the snow-affected NIRv values for which the NDSI > 0.1 [16]. The 5^th^ percentile of high-quality NIRv values observed within a five-year temporal window was used to replace the snow-affected observations in the NIRv time series [10]. Any remaining data gaps in the NIRv time series were filled using linear interpolation.

### 4.2 Smoothing the NIRv Time Series

**Fig. 3:**
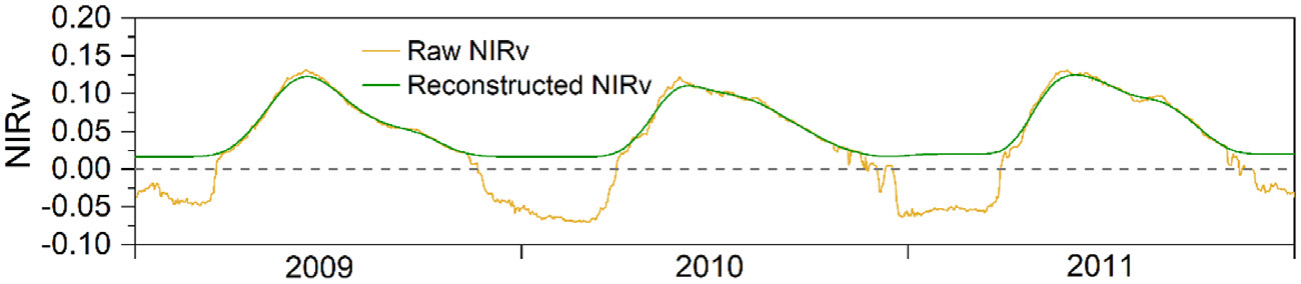
An example of the reconstructed near-infrared reflectance of vegetation (NIRv) time series.

Droughts can lead to complex seasonal NIRv profiles of drylands in Central Asia. Here, I applied a median filter to detect spikes in the NIRv time series [17]. If the absolute difference between the raw and the filtered NIRv values (11-day temporal window) was greater than 2.5 times the standard deviation of the raw NIRv values within a 21-day window, the raw NIRv value was replaced with the filtered value. The stationary wavelet transform (SWT), a local filtering method, was then applied to further reduce the noise in the NIRv time series [18,19]. SWT was performed on the NIRv time series at a decomposition level of 5 using the Biorthogonal 1.1 wavelet. The SWT decomposed the NIRv time series into an approximation signal and five detailed signals. Typically, the detailed signals contain information on high-frequency fluctuations in the NIRv time series, which may be considered noise [19]. Here, all detailed signals were eliminated to obtain a smooth NIRv time series [20]. An example of the final NIRv time series reconstructed through the two aforementioned steps is shown in Fig. 3.

### 4.3 Retrieving the LSP Metrics

The LSP metrics were estimated annually within a 14-month temporal window. For example, when retrieving the LSP metrics for 2005, NIRv data from 11/2/2004 to 12/31/2005 were used. A single growing season was considered for the LSPCA dataset. The maximum NIRv peak (NIRv_PEAK) and the corresponding date (POS) were initially detected. POS was assumed to occur prior to DOY 258. The following steps were performed if the detected NIRv_PEAK exceeded 0.015. First, the seasonal minimum NIRv values before and after POS were detected. Then, the seasonal amplitudes of the NIRv for the vegetation green-up (AMP_G) and senescence (AMP_S) phases within the temporal window were calculated. Finally, the thresholds set to 20% of AMP_G and AMP_S were applied to retrieve the SOS and EOS, respectively [7]. If the detected POS occurred prior to DOY 32, all LSP metrics for the corresponding growing season were masked, as the NIRv time series for that growing season may be abnormal.

## Limitations

In vegetated drylands, droughts can result in extremely low vegetation productivity and abnormal seasonal NIRv profiles. Consequently, cases of missing data can occur in the 23-year time series of an LSP metric, particularly for desert vegetation pixels. In such cases, a long-term trend analysis of the LSP metrics for a specific pixel cannot be carried out.

## Ethics Statement

The author confirms that the current work does not involve human subjects, animal experiments, or any data collected from social media platforms.

## CRediT authorship contribution statement

**Chao Ding:** Conceptualization, Methodology, Software, Resources, Data curation, Visualization, Writing – original draft.

## Data Availability

A land surface phenology dataset for Central Asia derived from MODIS NIRv time series (Original data) (Mendeley Data).
